# Retinoic acid enhances skeletal muscle progenitor formation and bypasses inhibition by bone morphogenetic protein 4 but not dominant negative β-catenin

**DOI:** 10.1186/1741-7007-7-67

**Published:** 2009-10-08

**Authors:** Karen AM Kennedy, Tammy Porter, Virja Mehta, Scott D Ryan, Feodor Price, Vian Peshdary, Christina Karamboulas, Josée Savage, Thomas A Drysdale, Shun-Cheng Li, Steffany AL Bennett, Ilona S Skerjanc

**Affiliations:** 1 Department of Biochemistry, Microbiology and Immunology, University of Ottawa, Ottawa, Ontario, Canada; 2 Neural Regeneration Laboratory and Ottawa Institute of Systems Biology, University of Ottawa, Ottawa, Ontario, Canada; 3 Department of Biochemistry, Medical Sciences Building, The University of Western Ontario, London, Ontario, Canada; 4 Ottawa Health Research Institute, Molecular Medicine Program, Ottawa, Ontario, Canada; 5 Department of Pediatrics and Physiology and Pharmacology, The University of Western Ontario, Children's Health Research Institute, London, Ontario, Canada

## Abstract

**Background:**

Understanding stem cell differentiation is essential for the future design of cell therapies. While retinoic acid (RA) is the most potent small molecule enhancer of skeletal myogenesis in stem cells, the stage and mechanism of its function has not yet been elucidated. Further, the intersection of RA with other signalling pathways that stimulate or inhibit myogenesis (such as Wnt and BMP4, respectively) is unknown. Thus, the purpose of this study is to examine the molecular mechanisms by which RA enhances skeletal myogenesis and interacts with Wnt and BMP4 signalling during P19 or mouse embryonic stem (ES) cell differentiation.

**Results:**

Treatment of P19 or mouse ES cells with low levels of RA led to an enhancement of skeletal myogenesis by upregulating the expression of the mesodermal marker, Wnt3a, the skeletal muscle progenitor factors Pax3 and Meox1, and the myogenic regulatory factors (MRFs) MyoD and myogenin. By chromatin immunoprecipitation, RA receptors (RARs) bound directly to regulatory regions in the Wnt3a, Pax3, and Meox1 genes and RA activated a β-catenin-responsive promoter in aggregated P19 cells. In the presence of a dominant negative β-catenin/engrailed repressor fusion protein, RA could not bypass the inhibition of skeletal myogenesis nor upregulate Meox1 or MyoD. Thus, RA functions both upstream and downstream of Wnt signalling. In contrast, it functions downstream of BMP4, as it abrogates BMP4 inhibition of myogenesis and Meox1, Pax3, and MyoD expression. Furthermore, RA downregulated BMP4 expression and upregulated the BMP4 inhibitor, Tob1. Finally, RA inhibited cardiomyogenesis but not in the presence of BMP4.

**Conclusion:**

RA can enhance skeletal myogenesis in stem cells at the muscle specification/progenitor stage by activating RARs bound directly to mesoderm and skeletal muscle progenitor genes, activating β-catenin function and inhibiting bone morphogenetic protein (BMP) signalling. Thus, a signalling pathway can function at multiple levels to positively regulate a developmental program and can function by abrogating inhibitory pathways. Finally, since RA enhances skeletal muscle progenitor formation, it will be a valuable tool for designing future stem cell therapies.

## Background

The initiation of skeletal myogenesis involves a complex interplay of signalling molecules secreted from the tissues surrounding the somite, including Wnt, Sonic hedgehog, and Bone morphogenetic proteins 4 (BMP4) [1-5]. Somites respond to the various signals by activating the expression of transcription factors that specify cells to the skeletal muscle lineage, including Pax3, Meox1 and Gli2 [6-10]. Commitment into skeletal myoblasts is dependent on the expression of the myogenic regulatory factors (MRFs), including MyoD, Myf-5, myogenin and myf-6/MRF4/herculin, and is regulated by factors in the dermomyotome [11,12].

P19 cells are pluripotent embryonal carcinoma (EC) cells, derived from mouse embryonic stem (ES) cells, that can differentiate into cardiac and skeletal muscle in a dimethylsulfoxide (DMSO)- and aggregation-dependent manner [13]. While cells grown in monolayer maintain their stem cell phenotype, the process of cellular aggregation initiates mesoderm induction, shown by the expression of Brachyury T [14]. Subsequent muscle development proceeds in the presence of DMSO. The order of transcription factors and signalling pathways for myogenesis in P19 cells appear to be similar to those during early embryogenesis. Thus P19 cells are a useful tool for examining *in vitro *myogenesis, potentially leading to novel mechanisms relevant to ES stem cell therapy.

Retinoic acid (RA) is a derivative of vitamin A and plays a crucial role in a wide variety of embryonic developmental processes [15]. In the embryo, the ability of RA to bind its receptors (retinoic acid receptors [RARs]/retinoid × receptors [RXRs]) is precisely controlled by regulating the availability of RA through proteins that synthesize RA, such as retinaldehyde dehydrogenase 2 (RALDH2), and those that metabolize RA, such as Cyp26, and other proteins that transport or bind RA.

Low levels of RA are known to enhance skeletal myogenesis in stem cells and myoblast cell lines [16-18]. RA can regulate MRF expression in myoblasts and chick limb [17-19], whereas RARs interact and synergize with MRFs [20]. However, the exact stage(s) at which RA functions to enhance skeletal myogenesis in a stem cell context has not been clearly defined.

Altered RA signalling in vertebrates affects body patterning, generating homeotic transformations and/or segmentation defects [21]. In *Xenopus *embryos, RA signalling regulates segmental patterning by promoting anterior segmental polarity and by positioning segmental boundaries [22]. In mice, RA coordinates somitogenesis and left-right patterning [23]. How the effect of RA on the somite impacts on the development of the myotome is not well understood.

In the canonical pathway, Wnt binds to cell surface receptors of the frizzled family, leading to the activation of Dishevelled and stabilization of cytosolic β-catenin [24]. In a simplified view, β-catenin enters the nucleus, binds the T-cell factor/lymphoid enhancer factor (TCF/LEF) family of transcription factors, and activates gene expression. Several studies have shown that exogenous Wnt and/or activated β-catenin can replace the dorsal neural tube in the induction of myogenesis in somite explant cultures [25]. A combination of Wnt and Shh signals regulates the expression levels of both β-catenin and Lef1 in the myotome prior to MyoD expression in the chick [26]. Further, β-catenin regulates the expression of Pax3 [27]. In P19 cells, a dominant negative β-catenin inhibits the expression of Pax3, Gli2, Meox1, MyoD, and abrogates myogenesis [8]. Finally, Wnt was shown to act directly on the Myf5 epaxial enhancer via β-catenin [28]. Therefore, there is strong evidence that Wnt signalling regulates specification and commitment into the skeletal muscle lineage. How Wnt signalling intersects with RA signalling during myogenesis is unknown.

BMP4 belongs to the TGF-β superfamily of peptide growth factors [29]. BMPs inhibit myogenesis in myoblast cell lines, limb micromass cultures, and developing somites [3,30,31]. Noggin signals are derived from the notochord and the somite. Noggin signalling is believed to counteract the inhibitory effects of BMP4 on the epaxial somite [32-34]. Further experiments using somite explants showed that relative levels of BMP4 and noggin regulated the activity of Pax3 to control the temporal and spatial activation of the MRFs [35]. Therefore, extensive studies have demonstrated the inhibition of embryonic skeletal myogenesis by BMP. How BMP signalling intersects with RA signalling is unknown.

The role of BMP in cardiomyogenesis has also been extensively studied. In *Drosophila*, the BMP homologue decapentaplegic protein (dpp) is secreted from the dorsal ectoderm and maintains tinman expression in the mesoderm [36]. Similarly, in chick BMP2 or -4 is expressed in tissues adjacent to the precardiac mesoderm and can induce Nkx2-5 and GATA-4 expression [37,38]. Conversely, disruption of BMP signalling with noggin or dominant negative receptors can prevent cardiomyogenesis in chick, *Xenopus*, ES, P19 and P19CL6 cells [37,39-45]. Therefore, BMP/dpp signalling is essential in controlling cardiomyogenesis.

Studies with embryonic stem and embryonic carcinoma cells have shown that RA inhibits cardiomyogenesis when added at an early stage [16,46,47] and enhances ES cell cardiomyogenesis when added at a late stage of differentiation [46,48]. RA can block myocardial gene expression, including XNkx2.5, in *Xenopus *embryos [49] and can alter cardiomyogenesis proliferation and patterning in other model systems [50-53]. Mice lacking various combinations of RXRs and RARs have shown that retinoids are required to prevent differentiation and support proliferation of ventricular cardiomyocytes [54,55]. RA deficiency in RALDH2 -/- mice alters second heart field formation [56,57]. Clearly, RA affects the timing and positioning of cardiomyogenesis at multiple levels and further studies are required to dissect out the role of RA at each step of development.

Here we investigate signalling events leading to the control of stem cell entry into skeletal and cardiac muscle lineages by RA, Wnt, and BMP4. We show that low levels of RA stimulate skeletal myogenesis by accelerating and increasing the expression of Wnt3a, Pax3, Meox1, and MRFs. This early and enhanced activation of skeletal muscle is refractory to inhibitory signals from BMP4 but not from a dominant negative β-catenin. Furthermore, low levels of RA inhibit stem cell differentiation into the cardiac muscle lineage, as shown by the absence of GATA-4 expression. The inhibitory activity of RA on cardiomyogenesis can be abrogated by the presence of BMP4. Therefore, BMP4 and RA function antagonistically to regulate each other's inhibition of entry into skeletal and cardiac muscle lineages, respectively. However, RA functions both upstream and downstream of Wnt signalling through β-catenin.

## Results

### RA inhibits cardiomyogenesis and enhances entry into the skeletal muscle lineage in P19 cells

**Figure 1:**
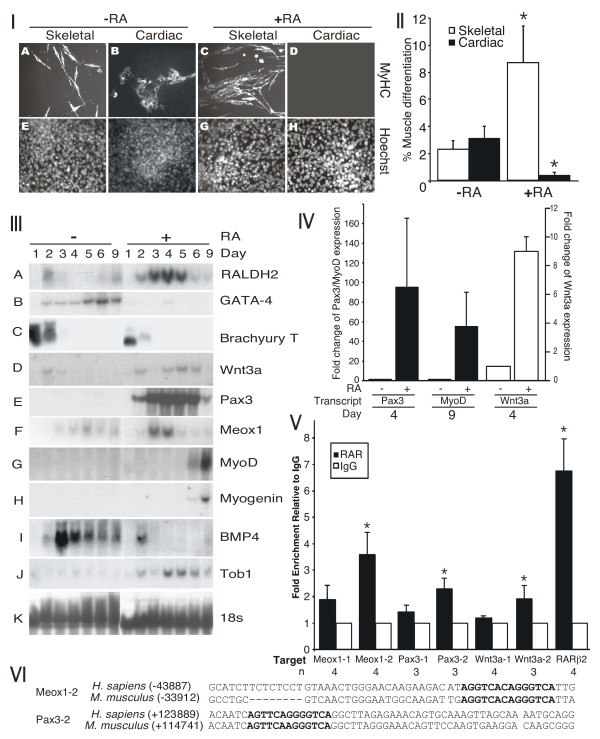
**Retinoic acid inhibits cardiomyogenesis and enhances skeletal myogenesis in P19 cells**. P19 cells were aggregated with 0.8% dimethylsulfoxide (DMSO) in the presence and absence of 30 nM RA. *Panel I*: Cells were fixed on day 9 for immunofluorescence with MF20 antibody (A-D) and counter stained with Hoechst dye (E-H). Magnification is 160x. *Panel II*: Cardiac (*n *= 3) and skeletal (*n *= 4) myogenesis were quantified by counting the number of MHC+ve myocytes as a percentage of the total. Average +/- standard error of mean (SEM) is shown and statistics were Student's t-test, *P < 0.05. *Panel III*: Total RNA was harvested for northern blot analysis on the days indicated and hybridized to the cDNAs on the right. Lanes are spliced from the same autoradiogram. *Panel IV*: Quantitative polymerase chain reaction (PCR) analysis was performed on day 4 of differentiation for Pax3 and Wnt3a transcript levels (*n *= 2) and on day 9 for MyoD levels (*n *= 4). Results were expressed as fold change of transcript levels in the presence compared to the absence of RA treatment. *Panel V*: Chromatin immunoprecipitation experiments were performed on day 2 P19 aggregates treated with DMSO/retinoic acid and analysed by real-time PCR using primers for sites within regulatory regions of the genes indicated. Average +/-SEM is shown, relative to IgG, and statistics were Student's *t*-test of each region compared to IgG, n = 3-4, *P < 0.05. *Panel VI*: The position and conservation of the Meox1-2 and the Pax3-2 retinoic acid response elements are shown.

Previous studies have shown that RA can inhibit cardiomyogenesis and enhance skeletal myogenesis [16,47], but the stage at which this occurs and the interaction with other signalling pathways have not been clearly defined. To investigate the mechanisms by which RA modulates myogenesis in P19 cells, various concentrations of RA, in the presence of DMSO, were examined. In agreement with previous results [47], it was found that skeletal myogenesis occurred in the presence of DMSO but not in its absence (summarized in Table 1). Furthermore, 3-30 nM of RA with DMSO was sufficient to block cardiac and enhance skeletal muscle development (data not shown). A time course of P19 cell differentiation was carried out in the presence of DMSO, with and without 30 nM of RA. Cells were fixed on day 9 for immunofluorescence and stained with an anti-myosin heavy chain (MyHC) antibody, MF20, which identifies MyHC in both cardiac and skeletal myocytes. Cardiac and skeletal myocytes can be distinguished by their morphology and time of appearance, with rounded cardiac myocytes appearing by day 6 and elongated, bipolar skeletal myocytes by day 9 [13]. Cells treated with RA did not differentiate into cardiac muscle, evidenced by the absence of rounded cardiac myocytes, expressing MyHC, compared to cells not treated with RA (Figure 1, panels IB and D). In contrast, significantly enhanced levels of bipolar skeletal myocytes were observed in the presence compared to the absence of RA (Figure 1, panels IA and C). Quantification of the number of cardiac and skeletal myocytes after treatment with RA showed that the fourfold increase in skeletal myocytes and eightfold loss of cardiac myocytes were statistically significant (Figure 1, panel II). Therefore, in agreement with others [58], RA inhibited cardiomyogenesis and enhanced skeletal myogenesis in P19 cells.

**Table 1:** Summary of gene expression changes in cell lines treated with and without DMSO and/or RA.

		**Cardiac**	**Skeletal Markers**				
**Cell line**	**Conditions**	**GATA-4 **	**Pax3**	**Gli2**	**Meox1**	**MRFs**	**Figure**

P19	-	-	-	-	-	-	Fig. 7 & Data not shown

P19	+DMSO	+	+	+	+	+	Figs. 1 & 4

P19	+DMSO&RA	-	+++	+	+++	+++	Figs. 1 & 4

P19 [β-cat/EnR]	+DMSO	N.D.	-	-	-	-	Figs. 3 & 4

P19 [β-cat/EnR]	+DMSO&RA	N.D.	+++	+++	-	-	Figs. 3 & 4

P19 [BMP]	-	-	-	-	-	-	Data not shown

P19 [BMP]	+DMSO	+	-	N.D.	-	-	Fig. 6, 7 & 8

P19 [BMP]	+DMSO&RA	+	+++	N.D.	N.D.	+++	Fig. 8

Mouse ES	-	+	-	N.D.	+/-	-	Fig. 2

Mouse ES	+RA	+/-	+++	N.D.	+++	+++	Fig. 2

P19 cell lines indicated on the right were aggregated under the conditions described and the induction of muscle marker gene expression was monitored.

(+++ = high expression; + = normal expression; +/- = partial expression, - = not expressed; N.D. = not determined)

In order to examine the molecular basis of the effects of RA on myogenesis, total RNA was harvested from a time course of cells differentiated with and without RA and subjected to northern blot analysis. Endogenous RALDH2 levels were enhanced on day 2 of the DMSO-induced differentiation in the absence of exogenous RA (Figure 1, panel IIIA), indicating that endogenous RA signalling could be involved at this stage of development [59]. RA-treated cells showed enhanced expression of RALDH2 from days 3-5 (Figure 1, panel IIIA). To determine at what stage cardiomyogenesis was inhibited by RA, expression of the cardiomyoblast gene GATA-4, was examined [60]. Northern blot analysis revealed a lack of induction of GATA-4 transcripts in cells treated with RA (Figure 1, panel IIIB), consistent with the interpretation that under these conditions RA inhibits commitment into the cardiac muscle lineage.

In order to determine at which stage skeletal myogenesis was enhanced, the expression of genes expressed in the primitive streak (Brachyury T and Wnt3a), the dermomyotome (Pax3 and Meox1), and skeletal myoblasts (MyoD and myogenin) was examined. Mesoderm induction occurred in the presence of RA, shown by the expression of Brachyury T (Figure 1, panel IIIC). The levels of Brachyury T appear to be slightly decreased with RA, consistent with the increasing differentiation of mesodermal progenitors. In contrast to the results with GATA-4 expression, Wnt3a (days 4-6), Pax3 (days 2-9), Meox1 (days 3-4), MyoD (days 6-9) and Myogenin (days 6-9) transcripts were upregulated with RA treatment (Figure 1, panels IIID-H), which is consistent with an increase in skeletal myogenesis. Quantitative polymerase chain reaction (Q-PCR) was used to quantify the levels of Pax3, MyoD and Wnt3a transcripts, showing a 9-95-fold upregulation of these factors with RA treatment (Figure 1, panel IV). Hence, RA enhances skeletal myogenesis by upregulating Wnt3a, Pax3 and Meox1 (summarized in Table 1).

Since BMP4 is known to inhibit skeletal and enhance cardiac myogenesis [61], and RA has been shown to inhibit BMP4 expression in the limb forebud [62], we examined the expression of BMP4 transcripts in the presence and absence of RA (Figure 1, panel III- I). The endogenous levels of BMP4 were down-regulated in RA-treated cells relative to the controls from days 3-9. Finally, we examined the expression of the transducer of ErbB2 (Tob1), an intrinsic inhibitor of BMP signalling [63,64]. Tob1 was upregulated by RA from days 2-9 (Figure 1, panel III-J). Thus, RA may function to enhance skeletal myogenesis and inhibit cardiomyogenesis in part by inhibiting BMP4 expression and/or function.

To identify direct chromatin targets of RAR binding, we used multiple sequence local alignment and visualization  to find RA response elements (RARE) sequences (defined as two tandem repeats of the RGKTCA element in DR1-7 arrangements) located within +/- 100 Kb of the start site for Wnt3a, Pax3 and Meox1. While all three genes contained RARE sequences, only one of the two Meox1 sites (Meox1-2) and one of the two Pax3 sites (Pax3-2) were conserved between mouse and human, located in *Mus musculus *at -33869 bp and + 114735, respectively (Figure 1, panel VI). Chromatin immunoprecipitation experiments were performed on day 2 of differentiation, using an antibody that recognizes all RARs. We detected a significant 3.6-fold enrichment in chromatin fragments corresponding to the Meox1-2 site, and a significant 2.3-fold enrichment in chromatin fragments corresponding to the Pax3-2 site compared to immunoprecipitation with a rabbit IgG as a negative control (Figure 1, panel V). We also tested several of the non-conserved RAREs identified upstream of Wnt3a. One of these RAREs (Wnt3a-2 at position -36625) showed a significant 1.9-fold enrichment. Several non-conserved RARE sequences in Wnt3a, Meox1, or Pax3 genes were not significantly associated with immunoprecipitated RARs, whereas a known site in RARβ2 was associated, as expected [65]. Thus, RARs bind to conserved elements in the Meox1 and Pax3 genes as well as to one non-conserved element upstream of Wnt3a in a population of differentiating P19 cells, indicating that RA functions both upstream and downstream of Wnt3a signalling.

### RA enhances skeletal myogenesis in mES cells

**Figure 2:**
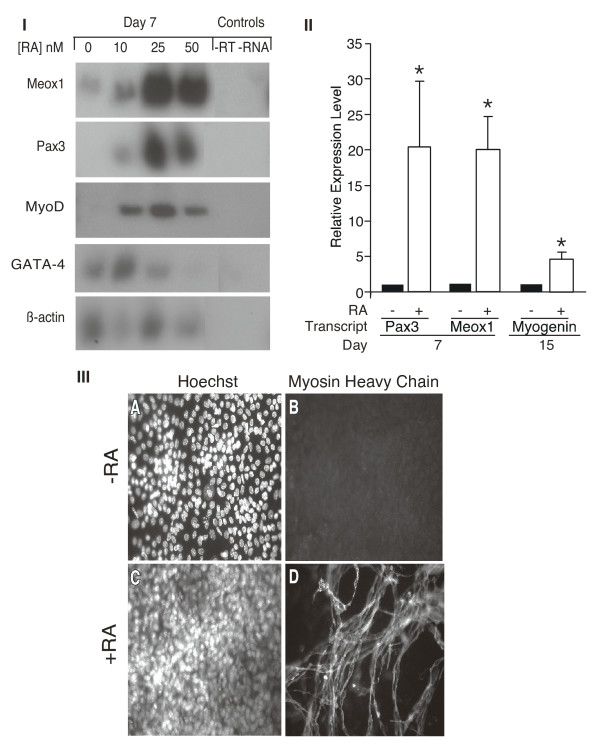
**Mouse embryonic stem (mES) cells differentiate into skeletal muscle in response to retinoic acid (RA)**. mES cells were aggregated in hanging drops for 2 days, cultured in suspension for a further 5 days with increasing concentrations of RA, and transferred to tissue culture dishes. *Panel I*: RNA was harvested from day 7 cultures and subjected to reverse transcriptase- polymerase chain reaction (PCR) followed by Southern blot analysis with the probes indicated on the left. *Panel II*: RNA was harvested from days 7 and 15 after differentiation with or without 25nM retinoic acid and examined by quantitative PCR analysis. The expression levels are expressed as fold increase in the presence, compared to the absence of RA, as the mean and standard error, *n *= 3. Statistics were Student's *t*-test, *P < 0.05. *Panel III*: On day 20 of differentiation, cells were fixed and reacted with Hoechst dye to detect nuclei (A and C) and with MF20 antibody to detect muscle (B and D). Magnification is 400×.

To determine if our results in P19 cells were applicable to mouse embryonic stem (mES) cells, we differentiated mES cells in hanging drops for 2 days and in suspension culture for an additional 5 days, the latter with 0-50 nM RA. After re-plating in tissue culture dishes, cells were harvested on days 7 and 15 for RNA and fixed on day 20 for immunofluorescence. Examination of RNA by RT-PCR indicated increasing transcript levels of Meox1, Pax3, and MyoD in the presence of retinoic acid, peaking at 25 nM (Figure 2, panel I). Quantification of gene expression changes by Q-PCR indicated statistically significant 20-fold increases in Pax3 and Meox1 expression on day 7 and a significant fivefold increase in myogenin on day 15 (Figure 2, panel II). Finally, skeletal myocytes were not observed on day 20 in the absence of RA, as shown by the lack of bipolar cells reacting with MF20 (Figure 2, panel III-B). In the presence of 25 nM RA, MyHC^+ve ^bipolar skeletal myocytes were visible (Figure 2, panel IIID). Thus, similar to the findings in P19 cells, RA treatment of mES cells results in the enhancement of skeletal muscle progenitor formation, shown by the increase in expression of Pax3, Meox1, MyoD, and myogenin (summarized in Table 1).

### RA cannot bypass the inhibition of skeletal myogenesis by a dominant negative β-catenin

**Figure 3:**
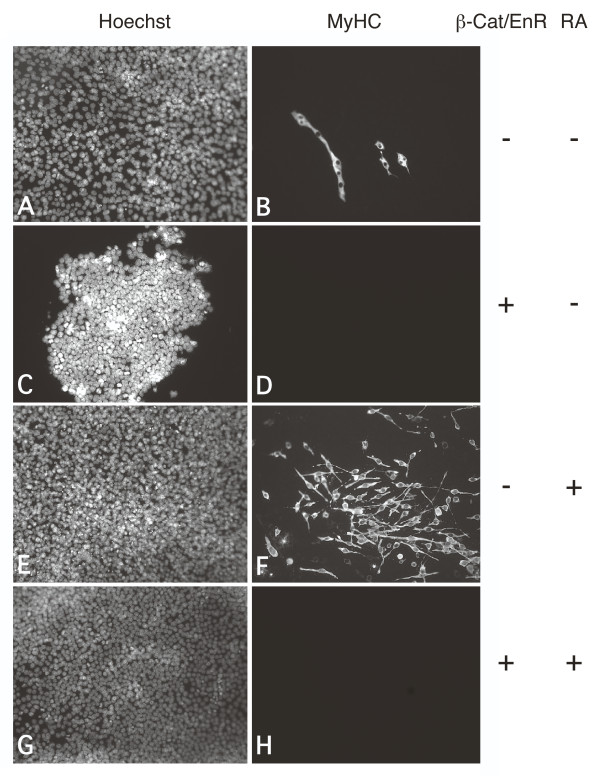
**Retinoic acid (RA) cannot override the inhibition of skeletal myogenesis by β-Cat/EnR**. P19[control] (A, B, E, F) and P19[β-Cat/EnR] (C, D, G, H) cells were aggregated in the presence of 0.8% dimethylsulfoxide (DMSO) with (E-H) and without (A-D) 10 nM RA. Cells were fixed on day 9 of differentiation for immunofluorescence with MF20 antibody (B, D, F, H) and counter stained with Hoechst dye (A, C, E, G). Magnification is 160×.

Since earlier studies have shown that Wnt signalling, via β-catenin, activates Pax3, Gli2 and Meox1 expression, inducing skeletal myogenesis in P19 cells [8], we were interested in determining how the Wnt signalling pathway intersects with RA signalling. Furthermore, a dominant negative β-catenin, with the transcriptional activation domain replaced by an engrailed repressor domain (β-Cat/EnR), inhibited skeletal myogenesis in P19 cells [8]. This dominant negative approach identifies genes bound by β-catenin and their downstream targets. In order to determine if RA can bypass this inhibition, P19 cells expressing β-Cat/EnR were differentiated in the presence of DMSO, with and without RA and compared to control P19 cells. Cultures were fixed on day 9 and examined by immunofluorescence with MF20. In agreement with previous results [8], the overexpression of β-Cat/EnR resulted in the loss of skeletal myogenesis, shown by the lack of bipolar skeletal myocytes, compared to control cells (Figure 3D versus B). The addition of RA resulted in an increase in the number of skeletal myocytes observed in control cells (Figure 3F versus B), but not in P19 [β-Cat/EnR] cells (Figure 3H). Therefore, RA was not sufficient to circumvent the inhibition of skeletal myogenesis by β-Cat/EnR.

**Figure 4:**
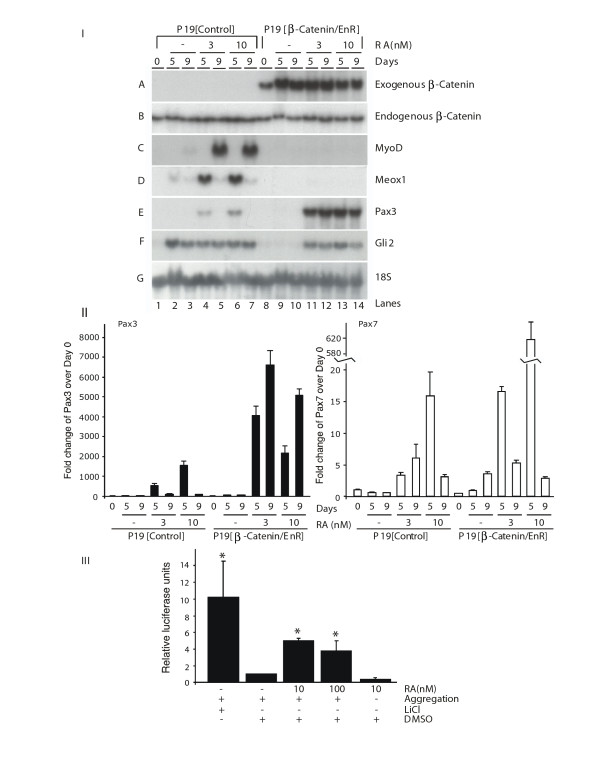
**Retinoic acid (RA) enhances the expression of Pax3/7 but not MyoD or Meox1 in the presence of β-Cat/EnR**. *Panel I*: P19[control] and P19[β-Cat/EnR] cultures were differentiated with 0.8% dimethylsulfoxide in the presence of 0, 3, and 10 nM RA. Total RNA was harvested and hybridized with the probes as indicated. *Panel II*: quantitative polymerase chain reaction analysis of Pax3 and Pax7 transcript levels, for each condition in Panel I, shown as one representative experiment performed in triplicate. *Panel III*: RA activates β-catenin in P19 aggregate but not monolayer cultures. P19 cells were transfected with the TOPFlash or FOPFlash reporter and treated with the compounds indicated in aggregated or monolayer cultures. Cells were harvested 24 hours later for luciferase assays (*n *= 2). Numbers represent the average +/- standard error of mean and statistics were Student's *t*-test, *P < 0.05.

To examine at which time point the RA enhancement of skeletal myogenesis was inhibited by β-Cat/EnR, RNA was harvested on days 0, 5 and 9 from P19 [control] and P19 [β-Cat/EnR] cultures differentiated with increasing amounts of RA. Northern blots showed the expression of exogenous β-Cat/EnR in the P19 [β-Cat/EnR] cells and not in P19 [control] cells (Figure 4, panel IA). Endogenous β-Catenin expression was constitutive, as expected (Figure 4, panel IB). In agreement with Figure 1, MyoD, Meox1 and Pax3 were upregulated with 3 and 10 nM RA in P19 [control] cells, compared to no RA treatment (Figure 4, panels IC - E, lanes 4-7 versus lanes 2-3). Gli2 was upregulated during myogenesis on days 5 and 9, in agreement with previous results [9], but was not further upregulated by RA (Figure 4, panel IF). Interestingly, while MyoD and Meox1 were no longer upregulated by RA in the presence of β-Cat/EnR (Figure 4, panels IC - D, lanes 11-14), Pax3 transcript levels were increased by RA treatment (Figure 4, panel IE, lanes 11-14). Myogenin and Myf-5 were also not upregulated in P19 [β-Cat/EnR] cells treated with RA (data not shown). The enhancement of Gli2 during myogenesis (Figure 4, panel IF, lanes 2-3) was abrogated in the presence of β-Cat/EnR (lanes 9-10), as shown previously [8]. This loss of Gli2 expression was reversed in the presence of RA (Figure 4, panel IF, lanes 11-14). Therefore, the RA enhancement of MyoD and Meox1 was abrogated in the presence of β-Cat/EnR, but not the enhancement of Pax3. Furthermore, the Gli2 expression became responsive to RA in the presence of β-Cat/EnR (summarized in Table 1).

Changes in gene expression for Pax3 and Pax7 were quantified by Q-PCR (Figure 4, panel II). RA treatment resulted in the increased expression of Pax3 and Pax7 in P19 [β-Cat/EnR] cells on days 5 and 9 (Figure 4, panel II). Thus, both Pax3 and Pax7 were upregulated by RA in the presence of β-Cat/EnR.

To further assess the relationship between RA and Wnt, the ability of RA to activate a β-catenin-responsive promoter - TOPflash - which contains 8 high mobility group (HMG) box sites, was examined using luciferase assays and compared to a mutated HMG box promoter, FOPflash [66]. Both 10 and 100 nM RA were sufficient to induce four- to fivefold increases in β-catenin activity in aggregated P19 cells (Figure 4, panel III). Interestingly, the activation by RA required cellular aggregation, since RA did not significantly enhance β-catenin function in monolayer cultures. Furthermore, no evidence was obtained of synergy or inhibition of RARβ on β-catenin activity using a β-catenin-responsive TOPflash reporter (data not shown), in contrast to previous reports of inhibition [67]. Thus RA can activate β-catenin function in DMSO-treated P19 aggregates, likely by enhancing β-catenin translocation to the nucleus.

### Induction of Pax3 by RA is not due to enhanced neurogenesis

**Figure 5:**
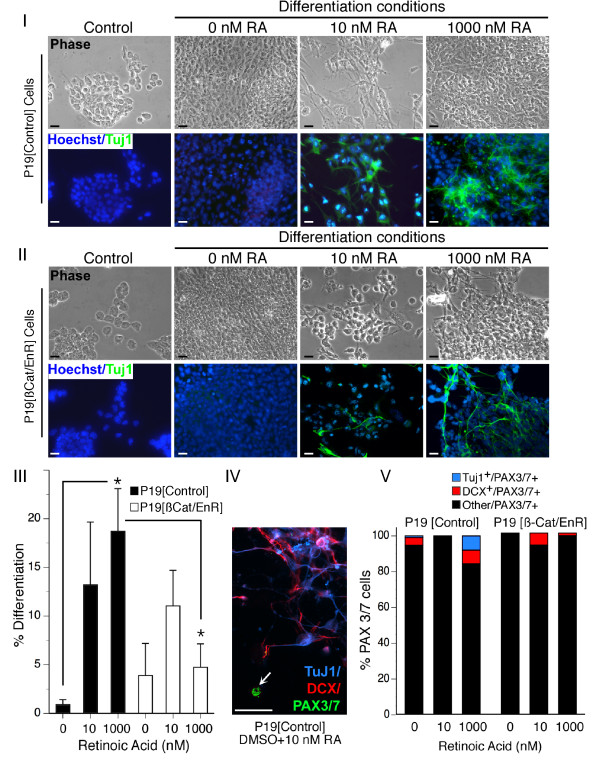
**PAX3/7 expression in P19 cultures treated with dimethylsulfoxide (DMSO) and 10 nM retinoic acid (RA) is indicative of skeletal myogenesis and not neurogenesis**. *Panel I*: P19[control] cells, undifferentiated in monolayer cultures (control) or differentiated with 1% DMSO, 1% DMSO+ 10 nM RA, or 1% DMSO + 1 μM RA for 9 days, were immunolabelled with anti-TuJ1 (green) to detect terminally differentiated neurons and stained with the nuclear marker Hoechst (blue). *Panel II*: P19[β-Cat/EnR] cells were treated as in Panel I. *Panel III*: Quantitative analysis of the percentage of Tuj1-positive cells was established, expressed as the percentage of the total cell number (*n *= 6-10). *Panel IV*: Immunofluorescent staining of PAX3/7 protein (green), committed neuronal precursors (doublecortin-positive, red), and terminally differentiated neurons (TuJ1-positive, blue) in triple-labelled P19[control] cultures treated with DMSO + 10 nM RA, demonstrating that PAX3/7-positive cells (arrow) are not neuronal precursors or neurons. *Panel V*: Quantitative analysis indicated that the overwhelming majority of PAX3/7-positive cells in all treatments were non-neuronal. Statistics were analysis of variance, *post*-*hoc *Bonferroni, *P < 0.05, Scale bars, 50 μm.

Since high levels of RA (1 μM) can induce neurogenesis in P19 cells and both Pax3 and Shh signalling via Gli2 can regulate neurogenesis in spinal cord and brain [68-70], the increase in Pax3 mRNA may not reflect skeletal myogenesis but, rather, result from an increase in neurogenesis. To test this hypothesis, we quantified the percentage of cells that differentiated to a neuronal phenotype in P19 [control] and P19 [β-Cat/EnR] cells under cardiac and skeletal myogenic conditions (1%DMSO), skeletal myogenic conditions (1%DMSO+10 nM RA), or neurogenic conditions (1%DMSO+ 1 μM RA) (Figure 5). Differentiation to a post-mitotic neuronal phenotype was identified by labelling of neuron-specific β-III tubulin using anti-Tuj1 antibodies. As expected, 1 μM RA triggered a significant neuronal differentiation compared to DMSO alone (Figure 5, panels I and III). However, neurogenesis with 10 nM RA was variable and was not significantly different compared to DMSO alone (Figure 5, panels I and III). Consistent with previous reports that the dominant-negative inhibition of β-catenin signalling reduces neurogenesis [71], we found that significantly fewer cells differentiated to Tuj1-positive neurons in P19 [β-Cat/EnR] cultures treated with 1 μM RA (Figure 5, panels II and III).

In order to determine whether Pax3/7 proteins were found in neurons, or in neural precursor cells, we used immunofluorescence to co-localize Pax3/7 proteins with neuronal markers (Figure 5, panels IV and V). Anti-Tuj1 antibodies were used to detect immature neurons and anti-double cortin (DCX) antibodies were used to detect committed neuronal precursor cells. DCX protein is expressed as early as 1 day after the initiation of neurogenesis in P19 cells and is the earliest known marker of neurogenesis [72]. To confirm that P19 cells do not express Pax3/7 following neuronal commitment, we quantified the percentage of Pax3/7-positive neuronal precursors and immature neurons in our day 9 cultures (Figure 5, panels IV and V). Under all conditions tested, the overwhelming majority of Pax3/7-positive cells were non-neuronal (Figure 5, panels IV and V). No Pax3/7-positive neurons were detected in P19 [control] cultures under conditions promoting skeletal myogenesis (DMSO + 10 nM RA) (Figure 5, panels IV and V). Taken together, these results provide strong evidence that enhancement of Pax3 and Gli2 expression by low concentrations of RA is indicative of increased skeletal myogenesis and not neurogenesis.

### Overexpression of BMP4 blocks skeletal myogenesis in P19 cells

**Figure 6:**
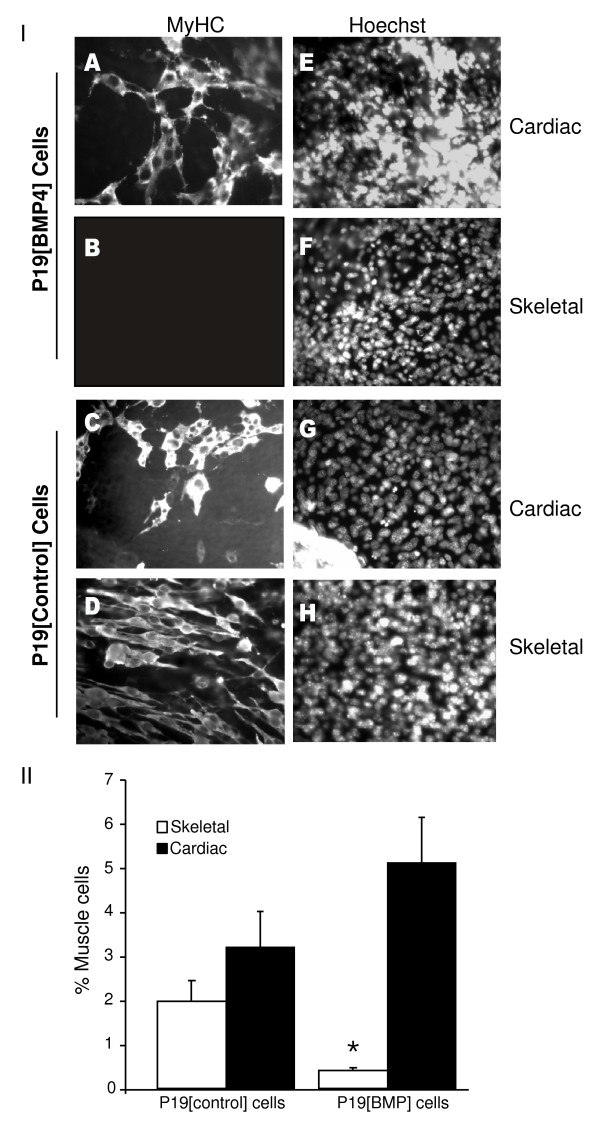
**BMP4 inhibits skeletal but not cardiac myogenesis**. *Panel I*: P19[BMP4] and P19[control] cells were aggregated in the presence of 0.8% dimethylsulfoxide (DMSO). Cells were fixed on day 9, stained with MF20 antibody (A-D), and counter-stained with Hoechst dye to show the nuclei (E-H). Magnification is 400x. *Panel II*: The number of MHC+ve cells were counted and the average +/- standard error of mean shown. Statistics were Student's *t*-test, *P < 0.05, *n *= 3.

Since RA enhances skeletal myogenesis, while downregulating BMP4 expression, we were interested in examining the interplay between RA and BMP4 in P19 cell myogenesis. In order to examine the effect of BMP4 on skeletal myogenesis in a stem cell context, stable cell lines expressing BMP4 were isolated and termed P19 [BMP4] cells. When aggregated in the absence of DMSO and examined for immunofluorescence with MF20, neither skeletal nor cardiac myogenesis occurred in P19 [BMP4] cells (data not shown). When cells were aggregated in the presence of DMSO, P19 [BMP4] cells differentiated into cardiac muscle (Figure 6, panel IA) but not skeletal muscle (Figure 6, panel IB). Under these conditions, control cells differentiated efficiently into both cardiac (Figure 6, panel IC) and skeletal muscle (Figure 6, panel ID). Furthermore, a similar inhibition of skeletal myogenesis was obtained when parental P19 cells were mixed with P19 [BMP4] cells in various ratios and aggregated in the presence of DMSO (data not shown). This indicated that BMP4 can function extracellularly. By counting the number of MHC+ve cells, skeletal myogenesis was inhibited an average of approximately fivefold in the presence of BMP4 and cardiomyogenesis was enhanced about 1.6-fold (Figure 6, panel II). Thus, overexpression of BMP4 in P19 cells is sufficient to block skeletal muscle and to slightly enhance cardiac muscle development.

### BMP4 inhibits skeletal muscle specification

**Figure 7:**
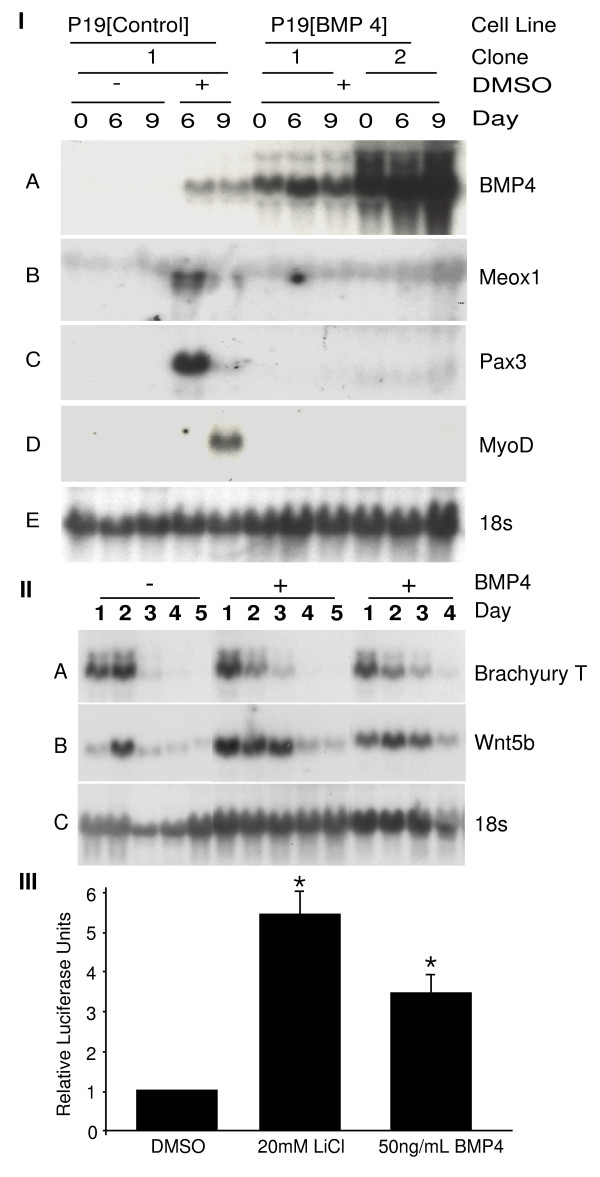
**BMP4 inhibits skeletal muscle specification**. *Panels I and II*: P19[BMP4] and P19[control] cells were aggregated in the presence of 0.8% dimethylsulfoxide (DMSO). P19[control] cells were also aggregated in the absence of DMSO to serve as negative controls. On days 0, 6 and 9 *(Panel I) *and days 1-5 (*Panel II*), total RNA was harvested for northern blot analysis and hybridized with the cDNAs indicated on the right. *Panel III*: P19 cells were transfected with the TOPFlash reporter, aggregated, and treated with the compounds indicated. Cells were harvested 24 hours later for luciferase assays (*n *= 2). Numbers represent the average +/- standard error of mean and statistics were Student's *t*-test, *P < 0.05.

**Figure 8:**
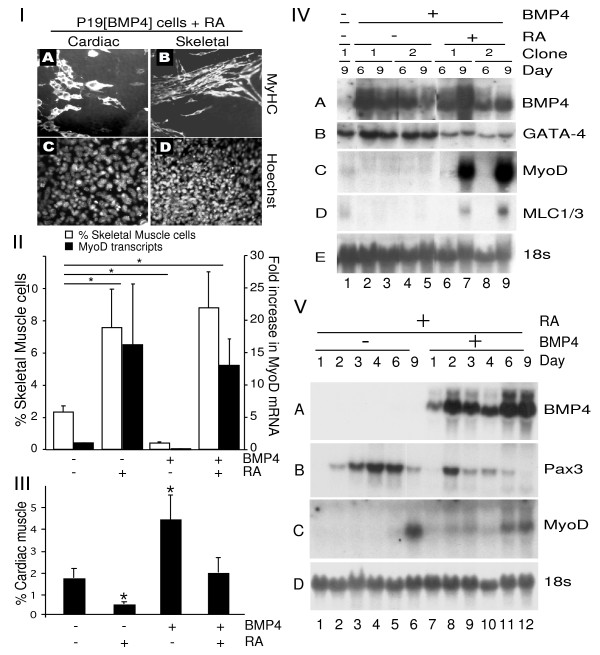
**Retinoic acid (RA) and BMP4 counteract each other's inhibition of skeletal myogenesis or cardiomyogenesis**. P19 cells were mixed with P19[BMP4] or P19[control] cells in the presence of 1% dimethylsulfoxide (DMSO), with or without RA. *Panel I*: P19[BMP4] cultures treated with RA were fixed on day 9 for immunofluorescence with MF20 antibody (A, B) and counter stained with Hoechst dye (C, D). Magnification is 160x. *Panel II*: Skeletal myogenesis was quantified for each condition by counting the number of myosin heavy chain^+ve ^bipolar skeletal myocytes, expressed as the percentage of total cells (white bars) and their standard errors (*n *= 3). MyoD transcript levels (black bars) were quantified by quantitative polymerase chain reaction and expressed relative to control cultures (*n *= 2), *P < 0.05. *Panel III*: Cardiomyogenesis was quantified as described for *Panel II*, *n *= 4. *Panel IV*: On days 6 and 9 total RNA was harvested for northern blot analysis and probed with the cDNAs indicated on the right. *Panel V*: A time course of P19[BMP4] and P19[control] cells aggregated in the presence of DMSO and RA. Total RNA was harvested for northern blot analysis on days 1-4, 6, and 9 and probed with the cDNAs indicated on the right.

In order to investigate at what point in the pathway BMP4 inhibited myogenesis, the expression patterns of skeletal muscle-specific markers in P19 [BMP4] and P19 [control] cells during DMSO-induced differentiation were compared. Total RNA was harvested on days 0, 6 and 9 for northern blot analysis. P19 [BMP4] cell lines expressed high levels of BMP4 (Figure 7, panel IA), but failed to express early markers of skeletal myogenesis such as Meox1 and Pax3 (Figure 7, panels IB - C) and late markers such as MyoD (Figure 7, panel ID) compared to P19 [control] cells. A 17-fold loss of MyoD transcript levels was detected by Q-PCR in the presence of BMP4 (Figure 8, panel II). These findings suggest that BMP4 inhibited an early stage of skeletal muscle development by preventing proper muscle specification (summarized in Table 1).

To determine if mesoderm induction, the stage prior to muscle specification, was affected by BMP4 expression, a time course of DMSO-induced differentiation was performed. P19 [BMP4] and P19 [control] cells were aggregated in the presence of DMSO and total RNA was harvested during the time course of differentiation for northern blot analysis. P19 [BMP4] and P19 [control] cell lines both expressed the mesoderm markers BrachyuryT and Wnt5b (Figure 7, panels IIA and B). Wnt5b expression levels appeared to be slightly increased. Finally, in order to compare the abilities of RA and BMP4 to enhance β-catenin activity, P19 cells were aggregated with DMSO in the presence and absence of BMP4 and the β-catenin-responsive TOPflash promoter was examined by luciferase assay. BMP4 was able to activate the β-catenin-responsive reporter in aggregated P19 cells treated with DMSO (Figure 7, panel III). Taken together, these results show that BMP4 interfered with skeletal myogenesis at a time point after mesoderm induction but before the specification of skeletal muscle.

### Reciprocal regulation of myogenesis by RA and BMP4

Since BMP4 was found to inhibit, and RA to enhance skeletal muscle specification in P19 cells, we wanted to determine if BMP4 could override the enhancement of skeletal myogenesis by RA, and vice versa for cardiomyogenesis. To this end, P19 cells were mixed with P19 [BMP4] or P19 [control] cells and aggregated in the presence of DMSO with or without RA. As expected, P19 cells, mixed with P19 [control] cells and aggregated in the presence of DMSO, differentiated readily into cardiac and skeletal muscle, as seen by positive MyHC staining. As shown in Figures 1 and 4, and quantified in Figure 8, RA inhibited cardiac myogenesis (3.6-fold), but not skeletal myogenesis, and BMP4 inhibited skeletal myogenesis (5.5-fold), but not cardiac myogenesis (Figure 8, panels II and III). However, P19 cells mixed with P19 [BMP4] cells and treated with RA differentiated efficiently into both cardiac and skeletal muscle (Figure 8, panels I-III), indicating that RA and BMP4 could antagonize each other's inhibitory activities.

The results of immunofluorescence were confirmed by RNA analysis. Cultures of P19 cells mixed with P19 [BMP4] cell lines contained high levels of BMP4 transcripts on days 6 and 9 (Figure 8, panel IVA, lanes 2-9). GATA-4 transcripts were present in cultures containing both BMP4 and RA, indicating that BMP4 abrogated the inhibition of cardiomyogenesis by RA (Figure 8, panel IVB, lanes 6-9). MyoD and myosin light chain1/3 (MLC1/3) transcripts were absent in P19 [BMP4], compared to P19 [control] cells (Figure 8, panel IVC - D, lanes 2-5 compared to 1). In contrast, P19 cells treated with BMP4 and RA robustly expressed MyoD and MLC1/3 transcripts (Figure 8, panels IVC and D, lanes 7 and 9). Quantification of MyoD transcript levels under the four conditions by Q-PCR was consistent with the results from counting skeletal myocytes (Figure 8, panel II). Therefore RA enhanced skeletal myogenesis and antagonized the inhibitory actions of BMP4 signalling.

Based on the inability of BMP4 to inhibit skeletal muscle development in the presence of RA, we predicted that the accelerated expression of Pax3 with RA treatment would still be observed in the presence of BMP4. To test this, we examined a time course of P19 cell differentiation in the presence of P19 [BMP4] cells and RA. Total RNA was harvested on days 1-4, 6, and 9 for northern blot analysis. Exogenous BMP4 transcripts were present in P19 [BMP4] cultures (Figure 8, panel VA). Pax3 and MyoD transcripts were detected in cells treated with RA alone in a similar expression pattern compared to cultures containing both RA and BMP4 (Figure 8, panels VB and C, lanes 1-6 compared to 7-12). These results are in contrast to the loss of Pax3 and MyoD expression shown in the presence of BMP4 alone (Figure 7). Therefore, the early enhancement of Pax3 expression by RA treatment still occurs in the presence of BMP4 (summarized in Table 1).

## Discussion

**Figure 9:**
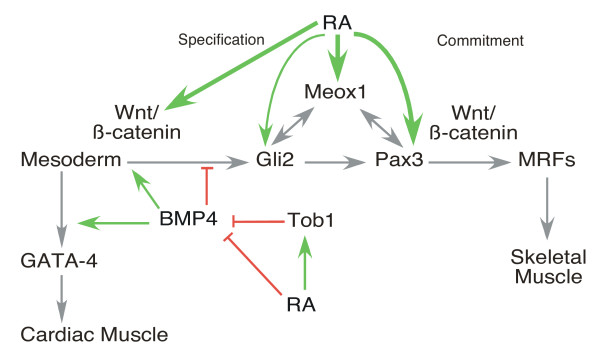
**Model of the intersection of retinoic acid (RA), Wnt, and BMP4 signalling during cardiac and skeletal muscle development**. BMP4 upregulates Wnt/β-catenin during mesoderm induction (green arrow) and blocks skeletal myogenesis by downregulation of Meox1, Pax3 and myogenic regulatory factor expression (red inhibition arrow). This inhibition can be reversed by RA, which enhances Tob1, Wnt3a, Pax3 and Meox1 expression, activates β-catenin and inhibits BMP4 expression (green arrows). RA receptors bind directly to the Wnt3a, Meox1, and Pax3 regulatory regions (bold green arrows). RA inhibits GATA-4 expression and cardiomyogenesis, likely by inhibiting BMP4 expression and function. Grey arrows indicate previous work [references [6,8-10,35,38,40,44,102,103].

We have examined the mechanism of P19 stem cell differentiation into skeletal muscle in response to RA, Wnt inhibition, and/or BMP4. We show that BMP signalling inhibits skeletal muscle specification, via the loss of Pax3 and Meox1, while RA enhances this step. Furthermore, RA can enhance skeletal myogenesis in the presence of BMP4 but not dominant negative β-catenin. RARs bound directly to RAREs in the upstream and downstream genomic regions of Meox1, Pax3 and Wnt3a. Both RA and BMP4 can activate the function of β-catenin in a reporter assay in aggregated, DMSO-treated, P19 cells. Thus, RA functions both upstream and downstream of Wnt3a signalling to enhance skeletal myogenesis. Inhibition by BMP4 can be bypassed by RA, implying that RA may function downstream of BMP4 or that BMP4 inhibition occurs by affecting RA signalling/generation (Figure 9).

In terms of cardiomyogenesis, RA signalling inhibits GATA-4 expression, resulting in the loss of cardiomyogenesis. RA blocks the expression of endogenous BMP4 and activates the expression of Tob1, which is an inhibitor of BMP function. The positioning of RA upstream of BMP4 expression and activity explains the ability of exogenous BMP4 to compensate for the low levels of BMP4 in the presence of RA, resulting in the enhancement of cardiomyogenesis (Figure 9).

We have shown previously that β-catenin is sufficient to induce skeletal myogenesis in P19 cells, by initiating skeletal muscle specification [8]. Here we show that RARs function as a positive regulator of Wnt signalling by binding directly to Wnt3a regulatory regions, upregulating Wnt3a transcript levels and activating of β-catenin. In other systems, such as F9/mouse ES cell differentiation or dorsal quail forebrain, RA can upregulate antagonists of the Wnt pathway, restrict Wnt expression, or inhibit β-catenin activity [73-75]. In contrast, RA upregulates the expression of Wnts during adult murine neurogenesis, or vertebrate limb induction [76,77]. Our observed activation of β-catenin by RA did not appear to involve synergistic interactions between RARs and β-catenin on a β-catenin-responsive promoter (data not shown). However, we have not ruled out that, once upregulated by RA signalling, β-catenin might synergize with RARs to activate RARE-responsive promoters, as described previously [78]. Interestingly, BMP4 treatment also resulted in the activation of the β-catenin-responsive promoter, which is not surprising given that BMPs and Wnts cooperatively pattern mesoderm [79]. It is likely that both RA and BMP4 act via canonical Wnt signalling to regulate both shared and distinct target genes, which change over time, dependent on combinatorial factors.

Pax3 expression is sufficient to initiate skeletal myogenesis in aggregated P19 cells [10] and it plays an important role in embryonic myogenesis [80]. RA accelerated Pax3 expression in P19 cells, likely by activating RARs bound directly to Pax3 regulatory sequences. Although Pax3 expression was not abrogated by the presence of β-Cat/EnR, and thus likely not bound by β-Cat/EnR, its expression alone was not sufficient to initiate skeletal myogenesis under these conditions. In contrast, although Meox1 is also a direct target of RARs, it was not upregulated in the presence of β-Cat/EnR which implies that β-Cat/EnR may have bound directly to the Meox1 regulatory sequences and inhibited its activation by RARs. Future studies will include a global analysis of RAR and β-catenin binding sites. The finding that RA cannot bypass the β-Cat/EnR inhibition implies that β-catenin and RARs bind to an overlapping, essential set of genes during myogenesis.

BMP4 inhibits skeletal myogenesis *in vitro *and *in vivo *[3,31,35,81]. In agreement with these studies, we show that BMP4 can inhibit the specification of P19 cells into the skeletal muscle lineage, shown by the loss of Pax3 and Meox1 expression. When combined, RA signalling was able to reverse the inhibition of Pax3 expression by BMP4. RA signalling enhances the expression of Tob-1, which is an inhibitor of BMP signalling. However, it is unlikely that RA functions solely by inhibiting BMP activity. For example, P19 cells expressing the BMP inhibitor noggin [44] did not show an enhancement/acceleration of skeletal myogenesis (M Jamali and I S Skerjanc, unpublished observations). Since RA can activate myogenic progenitor genes in the presence of BMP4, it likely functions to activate transcription downstream of the BMP inhibitory signal.

Low levels of RA inhibit cardiomyogenesis in P19 cells [16], although the mechanism has not been fully characterized. Here we show that RA inhibits the expression of GATA-4, preventing mesodermal cells from becoming committed to the cardiac muscle lineage. These results are similar to the finding that continued exposure to RA disrupts heart formation in *Xenopus *and zebrafish, although early in mesodermal patterning RA may increase the proportion of cardiac progenitors [49,53,82]. Low levels of RA may be redirecting pre-cardiac mesodermal cells into pre-skeletal mesoderm in P19 cells. Furthermore, RA may inhibit cardiomyogenesis via downregulation of BMP4 expression and upregulation of the BMP inhibitor Tob1. BMP4 functions to enhance cardiomyogenesis and can activate Nkx2-5 and GATA-4 expression [83]. The finding that exogenous BMP4 can override the inhibition of cardiomyogenesis by RA suggests that BMP4 functions downstream of the RA inhibition, or that BMP4 reduces the ability of RA to signal in cardiac muscle precursors.

In P19 and P19CL6 cells, noggin inhibits cardiomyogenesis, indicating that BMP signalling is essential [40,44]. However, we found that BMP4 overexpression was not sufficient to induce cardiomyogenesis in aggregated P19 cells without DMSO treatment, although a mild upregulation of cardiomyogenesis was observed with DMSO. Indeed, BMP4 during the aggregation stage may be inhibitory to cardiomyogenesis [84]. These results contrast with the role of sonic hedgehog (shh), which is sufficient to induce cardiomyogenesis in aggregated P19 cells without DMSO [85]. Shh may provide an earlier signal than BMP4 in the cascade of events leading to cardiomyogenesis.

In other systems BMP and RA have also been shown to function antagonistically. For example, noggin enhanced RA function in chick chondrocyte maturation [86]. In contrast, RA induced the expression of BMP-signalling molecules and enhances BMP effects in chondrocytes [87,88]. RA can induce or inhibit BMP expression, depending on the context [62,89,90]. Finally, BMP2/4 can inhibit RA-induced neurogenesis in P19 monolayer cultures [91]. Given the high degree of complexity of these signalling pathways, further studies are required to delineate the cross-talk mechanisms involved.

## Conclusion

In conclusion, we have examined the roles of RA, BMP4 and canonical Wnt signalling in directing entry into the cardiac and skeletal muscle lineages. RA enhanced skeletal myogenesis by inhibiting BMP4 expression and function, while activating the expression of pre-skeletal mesoderm genes, and activating Wnt/β-Catenin signalling. RA inhibited cardiomyogenesis, likely by inhibition of BMP4 function. RA and BMP4 can each reverse the other's inhibition of myogenesis. Therefore, the precise balance of these signalling molecules is necessary to regulate specification into skeletal or cardiac muscle.

## Methods

### Plasmid constructs

The expression construct phosphoglycerate kinase (PGK)-BMP4 contains a 1.8 Kb EcoRI BMP4 cDNA fragment, containing the complete coding sequence of human BMP4 (Wyeth Pharmaceuticals, MA, USA), driven by the *pgk-1 *promoter. The empty PGK vector was used as control. The constructs PGK-Puro, B17, and PGK-Lac-Z were previously described [92].

### P19 Cell culture and isolation of stable cell lines

P19 embryonal carcinoma cells (American Type Culture Collection, VA, USA) were cultured as previously described [93] in α-minimum essential media supplemented with 5% cosmic calf serum (Hyclone, UT, USA) and 5% fetal bovine serum (Cansera, Rexdale, ON, Canada). P19 [β-cat/EnR] cells were isolated and described previously [8]. For P19 [BMP4] cells, stable cell lines were created using similar protocols. Briefly, P19 cells were transfected with the DNA constructs PGK-BMP4 or the empty PGK vector along with PGK-puro, PGK-LacZ and B17 with the aid of the *Fugene *6 transfection kit (Roche Diagnostics Canada, Quebec, Canada) as per manufacturer's instructions. Twenty-four hours after transfection, cells were grown under puromycin selection for 10 days. Individual colonies were analysed for BMP4 expression.

To examine the role of BMP4 in P19 cell differentiation, P19 cells, P19 [BMP4] cells, P19 [control] cells, 1:1 mixtures of P19 and P19 [BMP4] cells, and 1:1 mixture of P19 and P19 [control] cells were aggregated with or without 0.8-1% DMSO for 4 days in petri dishes [94]. Subsequently cells were transferred to tissue culture plates or gelatin coated cover slips and examined for differentiation on days 6 or 9.

To examine the role of RA in P19 cell differentiation, P19 cells, P19 [β-cat/EnR] cells P19 [BMP4] cells, P19 [control] cells, 1:1 mixtures of P19 and P19 [BMP4] cells, and 1:1 mixture of P19 and P19 [control] cells were aggregated with or without 0.8-1% DMSO in the presence of 0-30 nM all-trans retinoic acid. Stocks of retinoic acid were purchased and prepared every 3-4 months. Each new stock was titrated for optimal skeletal myogenesis.

### Immunofluorescence

For analysis of muscle-specific markers, cultures were treated as described previously [95] with MF20 [96]. Staining was visualized with a Zeiss Axioskop microscope and photographed with a Sony 3CCD camera. Images were formatted using Adobe Photoshop and Canvas software. Myogenic differentiation was quantified by counting five to 10 fields containing 100-200 cells.

For analysis of neuron-specific markers, cultures were plated on gelatin-coated coverslips and cultures were fixed with methanol at -20°C. Antigenic analysis was performed using mouse-anti-Tuj1 (Research Diagnostics, USA) guinea pig anti-DCX (Chemicon, USA) and Hoechst dye as a nuclear marker. To identify PAX3/7-positive cells, some cultures were also labeled with goat-anti-PAX3/7 (Invitrogen, Canada). Secondary antibodies were AMCA- or FITC-conjugated anti-mouse-IgG, Cy3-conjugated anti-guinea pig-IgG, (Jackson ImmunoResearch Laboratories, USA) and Alexa-488 conjugated anti-goat IgG (Invitrogen, Canada) where appropriate. Immunofluorescence was evaluated using OpenLab Software, version 3.4 (Improvision, MA, USA) on a Leica DMXRA2 microscope equipped for epifluorescence. Antigen-positive cells were counted in three to 10 fields in triplicate samples performed over two independent experiments and data expressed as a percentage of the total cell number (*n *= 6-10). Counts were performed by two investigators blind as to the treatment conditions of each field.

### Northern blot analysis

Cells were harvested for RNA extraction using the Urea/lithium chloride method [97] as previously described [98]. The cDNA fragments used to probe for MLC1/3 and MyoD [47] and for BMP4, GATA4, Pax3, Meox1, Myogenin, BrachyuryT, Wnt5b and 18s have been described previously [10]. A 1.8 Kb Kpn1/Mlu1 full length human cDNA fragment was used to probe for Tob1 (GeneBank Accession Number: BC031406) and a 400 bp HindIII fragment for mouse RALDH2 (GI Number 31982069).

### mES cell culture and differentiation

J1 or D3 mES cells were maintained in 15% fetal bovine serum in the presence of leukemia inhibitory factor (LIF). Differentiation was induced by aggregating cells in the absence of LIF. Cells were aggregated in hanging drops containing 800 cells for 2 days and in suspension for a further 5 days. Aggregates were treated with increasing concentrations of retinoic acid from day 2 to day 5 or 7 of differentiation. On day 7 of differentiation, cells were plated on tissue culture plates and allowed to differentiate for a further 13 days. The efficiency of myocyte formation was enhanced by plating at a high cell density. Experiments were performed at least twice with each cell line with similar enhancement of skeletal myogenic precursors.

### PCR Analysis

For RT-PCR southern analysis, total RNA was harvested from day 7 mES cells and 0.8 *μ*g was subjected to reverse transcription (Qiagen, Mississauga, Canada). The resultant cDNA was subjected to PCR with primers specific for Pax3, Gata-4 and β-actin [85,99] and for MyoD and Meox1 [100] described previously. The PCR product was hybridized to DNA probes and visualized by autoradiography.

For quantitative real-time PCR (Q-PCR) expression analysis of mES cells, total RNA was harvested from differentiating cells using the RNeasy Micro kit (Qiagen, Mississauga, Canada). RNA harvested from P19 cells using the urea/lithium chloride extraction was further purified using the RNeasy Micro kit (Qiagen, Mississauga, Canada). The resultant cDNAs were used for Q-PCR, using protocols and primers for Pax3/7, Meox1, MyoD, myogenin and GAPDH as described previously [100]. Primers for β-actin are found in Table 1. mRNA levels were normalized to β-actin or glyceraldehyde-3-phosphate dehydrogenase levels for the corresponding day and subsequently to levels in untreated samples for the corresponding day.

### Promoter analysis

P19 cells were transfected with 6 μg of the super8 TOPFlash [101] or FOPFlash reporter plasmids (generous gifts from R Moon), as well as with 2 μg of Renilla Luciferase (Promega, WI, USA), using Fugene Transfection Reagent (Roche Applied Sciences, QC, Canada). Cells were either left in monolayer culture or aggregated in the presence of 1% DMSO, 20 mM LiCl, 1% DMSO with retinoic acid, or 1%DMSO with 50 ng/ml BMP4. Luciferase assays were conducted using the Dual Luciferase Reporter Assay System from Promega, 24 hours after treatment. Luciferase activity was normalized to *Renilla *activity. Data from samples treated with lithium chloride or RA were normalized to samples treated with DMSO alone.

### Chromatin Immunoprecipitation

Protein was cross-linked to DNA and chromatin was harvested as described previously [100] from three 150 mm dishes of day 2 P19 aggregates, treated with 1% DMSO and 10 nM RA. For immunoprecipitation, 2 μg of RAR antibody (Santa Cruz, CA, USA) or 2 μg of rabbit immunoglobulin G antiserum (Chemicon, MA, USA) was incubated with chromatin and the immune complexes were captured by addition of protein-G sepharose beads, as described [100]. After RNase A and proteinase K treatments, DNA was purified using Qiagen's PCR Purification Kit (Qiagen, ON, Canada). Relative enrichment of binding sites compared to the IgG negative control immunoprecipitation was analysed using SYBR green real-time PCR, as described above, using primers listed in Table 2.

**Table 2:** Oligonucleotide sequences of primers utilized for chromatin immunoprecipitation experiments.

**Target Gene**	**Target Sequence and Position**	**Forward primer**	**Reverse primer**
Meox1-1	AGTTCAAGCCTCA (-45995)	CATGAGTTCAAGCCTCAGCA	CCAGAGATACGCTTGGTGTC

Meox1-2	AGGTCACAGGGTCA(-33869)	GAGGCCTAGCTTCAGCTCCT	TGAAATGCCTGATCTGACACA

Wnt3a-1	TGAACTCATGACCC(-10844)	GAGGGAATCAAATCCCATTATAGA	GGCAGAACCTGTAGTCAGAAACT

Wnt3a-2	TGACCTTTTGACCT(-20576)	CAGGTATTGCCATCCAGGTT	GAGAATGCTCTGTGGGGTTC

Pax3-1	AGGTCAGGCTGGCTTCA(-97389)	ACAGGGTAAAACAATGTGTGGA	TTGAAGCCAGCCTGACCTAT

Pax3-2	AGTTCAAGGGTCA(+114735)	AGTGGAGCGCACCTCTGT	CTACAAACCCTTAATGACAAACG

RARβ2	GGTTCACCGAAAGTTCA(-319)	GGTTCACCGAAAGTTCACTCGCAT	CAGGCTCGCTCGGCCGATCCA

## Abbreviations

BMP: bone morphogenetic proteins; dpp: decapentaplegic protein; DMSO: dimethylsulfoxide; DR: direct repeat; EC: embryonal carcinoma; ES: embryonic stem; HMG: high mobility group; LEF: lymphoid enhancer factor; LIF: leukaemia inhibitory factor; mES: mouse ES; MRF: myogenic regulatory factors; MyHC: myosin heavy chain; PCR: polymerase chain reaction; PGK: phosphoglycerate kinase; Q-PCR: quantitative PCR; RA: retinoic acid; RALDH2: retinaldehyde dehydrogenase 2; RAR: RA receptor; RARE: RA response element; RXR: retinoid × receptors; Shh: sonic hedgehog; TCF: T-cell factor.

## Authors' contributions

KK carried out the initial analysis of the effect of RA on skeletal and cardiac myogenesis, with and without BMP4, and drafted the initial manuscript. TP carried out the analysis of the effect of RA on mouse ES cell differentiation and β-catenin function, the RAR ChIP experiments, and modified the manuscript. VM carried out the analysis of P19 [β-cat/EnR] cells. TP, SR and VP carried out the immunofluorescent analysis of neurogenesis in P19 [β-cat/EnR] cells. FP contributed experimentally to the mouse ES study. CK carried out the initial characterization of P19 [BMP4] cells. JS carried out the microarray analysis of RA treated cells, identifying Tob-1. TD participated in the interpretation of data for RA treated cells and the role of Tob-1 and revising the manuscript. S-CL contributed to providing lab space and reagents for acquisition of Q-PCR data. SALB conceived and designed the neurogenesis analysis. ISS conceived/designed the study and modified the manuscript. All authors read and approved the final manuscript.
